# Evaluation of Vitrectomy with Planned Foveal Detachment as Surgical Treatment for Refractory Diabetic Macular Edema with or without Vitreomacular Interface Abnormality

**DOI:** 10.1155/2018/9246384

**Published:** 2018-05-07

**Authors:** Ahmed M. Abdel Hadi

**Affiliations:** Ophthalmology, Faculty of Medicine, Alexandria University, Alexandria, Egypt

## Abstract

**Purpose:**

To evaluate the therapeutic efficacy of subretinal BSS injections done during vitrectomy for refractory diabetic macular edema (DME) resistant to other modes of treatment including previous vitrectomy.

**Materials and Methods:**

A prospective, interventional noncomparative case series in which cases had refractory DME with a central macular thickness (CMT) ≥ 300 *μ*m, despite previous anti-VEGF therapy (ranibizumab or bevacizumab with shifting to aflibercept). Some cases even received intravitreal triamcinolone acetonide injection, before attempting this solution. The study included group 1, surgically naïve eyes, and group 2, cases with persistent edema despite a previous vitrectomy (7 eyes (25%)). The cases were also divided into group a, eyes with normal vitreomacular interface, and group b, with abnormal vitreomacular attachment (VMA) (6 (21.4%)). The 1ry endpoint for this study was the change in CMT after 9–12 months from surgery. The 2ry endpoints were change in BCVA, recurrence of DME, and surgical complications.

**Results:**

The study included 28 eyes, 6 (21.4%) of which suffered from edema recurrence. The mean recorded CMT was 496 ± 88.7 *μ*m and 274.1 ± 31.6 *μ*m preoperatively and postoperatively, respectively. In all eyes, the preoperative mean BCVA in decimal form was 0.2 ± 0.11, which improved significantly to 0.45 ± 0.2. In the end, the CMT of groups 1 and 2 measured 239 *μ*m and 170.8 *μ*m, respectively (*p* = 0.019). The preoperative BCVA in groups 1 and 2 was 0.16 ± 0.07 and 0.37 ± 0.14, respectively, which improved to a mean of 0.34 ± 0.09 and 0.7 ± 0.16 postoperatively, respectively (*p* = 0.185).

**Conclusion:**

Vitrectomy with a planned foveal detachment technique was shown to be a promising solution for refractory DME cases with rapid edema resolution. CMT was shown to improve more in eyes where conventional vitrectomy was not attempted. Moreover, cases with VMA resistant to pharmacotherapy was shown to respond well to this technique. The study has been registered in Contact ClinicalTrials.gov PRS Identifier: NCT03345056.

## 1. Introduction

Many therapeutic options exist for diabetic macular edema (DME)—the leading cause of visual diminution in patients with diabetic retinopathy (DR). Since 2010, antivascular endothelial growth factors (anti-VEGF) have become the gold standard for DME treatment, replacing macular laser photocoagulation [1, 2].

Many eyes respond favorably to anti-VEGF agents; nevertheless, some do not achieve optimal edema control, and this group is referred to as refractory DME. The prevalence of refractory DME is estimated to be up to 50% [1], constituting a large unmet defect in DME management. Switching from one anti-VEGF agent to another is a viable first step for resistant DME management [3]. In addition, corticosteroids are considered by many researchers as the main therapy for DME refractory to anti-VEGF treatment, due to their multimodal actions [4]. Despite these strategies, resistant DME cases still exist.

Surgery is thought to play a role in nontractional cases, allowing a more efficient clearance of VEGF and other cytokines from the retina and allowing a better oxygen access from the anterior segment to the retina, thereby reducing DME [5]. In addition, the presence of a vitreoretinal interface abnormality (VRA) reduces the therapeutic effect of anti-VEGF agents in patients with DME. These agents may alter the balance between angiogenic and fibrotic growth factors in patients with diabetic retinopathy, termed the angiofibrotic switch, which can result in increased retinal traction in some patients with proliferative diabetic retinopathy (PDR) prior to surgery [6]. Vitrectomy can relieve this tractional component and can result in resolution of the edema [7].

Improvement of the condition of the retina after vitrectomy takes time, and during that time, the photoreceptor cells may become permanently damaged [8–11] by the chronic macular edema leading to poor visual prognosis [12]. Furthermore, recent optical coherence tomography (OCT) observations show that a shorter time from the onset of DME to its resolution is the major factor affecting the integrity of the ellipsoid zone and a good visual outcome [13, 14], indicating the importance of rapid resolution of DME after vitrectomy.

Morizane et al. evaluated the therapeutic efficacy of subretinal balanced salt solution (BSS) injections in conjunction with conventional vitrectomy for treating diffuse DME. They demonstrated that this technique is effective for rapid resolution of diffuse DME resistant to anti-VEGF therapy and for the improvement of visual acuity [15]. Their study did not evaluate the usefulness of this technique in cases with vitreomacular interface abnormality resistant to intravitreal pharmacotherapy. Intravitreal corticosteroids were also not tried in their cohort of resistant cases, because various methods for administering steroids, including dexamethasone intravitreal implants, were not approved in Japan at the time. Therefore, they used sub-Tenon injection of triamcinolone acetonide in their study [16].

The present study is aimed at evaluating the therapeutic efficacy of subretinal BSS injections in conjunction with conventional vitrectomy for refractory DME resistant to more than one anti-VEGF agent, intravitreal corticosteroids, and to previous vitrectomy.

## 2. Materials and Methods

This study was a prospective, interventional noncomparative case series. The author adhered to the tenets of the Declaration of Helsinki. All patients were informed about the risks and benefits of the surgery, and written consent was obtained after thorough explanation of the procedure in clear simple words. The study was approved by the Institutional Review Board and the Ethics Committee at the Faculty of Medicine, Alexandria University.

Twenty-eight eyes of 28 patients with DME resistant to anti-VEGF and corticosteroid (Cst) therapy were included in this study. Some had already undergone pars plana vitrectomy for refractory DME. In all cases, vitrectomy was performed with subretinal injection of BSS between November 2015 and November 2017.

The inclusion criterion for eyes with refractory DME was a central macular thickness (CMT) of more than 300 *μ*m despite undergoing anti-VEGF therapy (5-6 monthly injections of ranibizumab (IVR) or bevacizumab (IVB) with shifting to aflibercept (IVA) for additional three injections). Some cases received Cst injection as well, before attempting this surgical solution in the form of intravitreal triamcinolone acetonide (1 or 2 injections) three months apart. All cases were psuedophakic. Cases subjected to conventional vitrectomy with internal limiting membrane (ILM) peeling were also enrolled in the study.

They were analysed after subdivision into group 1, including cases in which vitrectomy was not attempted, and group 2, including cases with persistent edema despite a previous vitrectomy (performed at least 6 months before the intervention). The cases were also divided into two groups: group a with normal vitreomacular interface (VMI) (defined as the absence of either perifoveal vitreoretinal attachment within 2500 *μ*m of the foveal center or hyperreflective inner retinal band), group b with vitreomacular abnormality (VMA) in the form of ERM (defined as a hyperreflective inner retinal band with or without associated retinal inner surface plication).

The major exclusion criteria were (1) the presence of apparent retinal pigment epithelium (RPE) atrophy at or near the macula; (2) the presence of proliferative diabetic fibrovascular membranes threatening or at the macula; (3) the presence of diabetic optic atrophy; and (4) the presence of neovascular glaucoma.

All patients underwent complete ophthalmologic examinations with special emphasis on best-corrected visual acuity (BCVA) using the 6 m Landolt C acuity chart (converted to decimal) and indirect and contact lens slit lamp biomicroscopy. Spectral domain or swept source OCT (Cirrus; Carl Zeiss Meditec Inc., Dublin, CA; Spectralis; Heidelberg Engineering GmbH, Heidelberg, Germany) was used to examine all eyes before surgery and at 1 month and at the final visit after surgery. Central retinal thickness was defined as the distance between the inner surface of the RPE and the inner surface of the neurosensory retina at the macula. All patients were followed up for at least 10 months.

### 2.1. Data Analysis

To evaluate the surgical outcomes, preoperative and postoperative CMT and BCVAs of both groups (1, 2) and (a, b) were compared using paired tests. Significance was considered starting at a cut-off *p* value of 0.05. All statistical analyses were performed using SPSS for Windows, version 22.0 (SPSS Inc., Chicago, IL). Quantitative data are presented as mean ± standard deviation, while qualitative data are represented in number and percentage.

### 2.2. Surgical Technique

The surgery was performed using a 23-gauge, transconjunctival, microincision vitrectomy system. After core vitrectomy, posterior hyaloid detachment was attempted with the vitrectomy cutter in the suction mode. We then stained the ILM with dual stain (MembraneBlue-Dual, DORC International), which contains a combination of 0.15% trypan blue, 0.025% Brilliant Blue G (BBG), and 4.00% polyethylene glycol (PEG). It was injected under air and left there for 30 seconds. Subsequently, the ILM peeling was attempted and peripheral vitrectomy was carried out as the peripheral residual vitreous was more evident after the dual stain application. We then injected 0.3–0.5 ml of BSS into the subretinal space to detach the fovea, ensuring that the foveal detachment covered the entire area with DME. This injection of BSS was performed at the site where the ILM had been removed using a 38-gauge cannula (MedOne Surgical Inc., Sarasota, FL) with a pressure of 4 to 6 psi (viscous fluid control system, Alcon Laboratories, Fort Worth, TX) [17] (Video 1).

In cases with VMI abnormality, the EMM was peeled using an end-gripping 23-gauge forceps after staining with dual stain, which stains both the ILM and the ERM. Then, ILM peeling was attempted with the 23-gauge end-grasping forceps (Rumex International Co., USA). Subretinal injection of BSS was done as mentioned above (Video 2).

In eyes with persistent DME despite previous vitrectomy, staining was also done under air to ensure ILM removal above the entire area involved in the edema process and proper peripheral vitreous trimming before attempting subretinal BSS injection (Video 3).

### 2.3. Endpoints

The primary endpoint for this study was the change in CMT at the final visit (from 9–12 months after surgery). The secondary endpoints were change in BCVA at the final visit after surgery, recurrence of DME, and surgical complications. The state of the ellipsoid zone and the ELM (as shown by the preoperative OCT) was also compared to its appearance in the OCT taken during the final visit. Recurrence of DME was defined as an increase in CMT ≥ 10% of the least thickness attained during the period of follow-up, with concomitant drop of at least one line of BCVA.

## 3. Results

### 3.1. Preoperative Characteristics

The study included 28 eyes of 28 patients with a mean age of 53.1 ± 7.2 years. All eyes had CME, with 13 eyes (46.4%) suffering from neurosensory detachment (NSD), while only 6 eyes (21.4%) had vitreomacular interface abnormality (VMA) in the form of a fine epimacular membrane. Thirteen eyes (46.4%) received IVB followed by 3 IVA injections before including them in this study, while 16 eyes (57.1%) received preoperative IVR followed by 3 IVA before rendering them refractory and including them in the study. CST was given in 12 eyes (42.9%) after failure of either protocol of anti-VEGF to decrease CMT.

As regards the preoperative OCT finding, preoperative ellipsoid zone was intact in 13 eyes (46.4%) and disrupted in the rest of the included eyes. The preoperative ELM was intact in 12 eyes (42.9%) preoperatively. In 7 eyes (25%), a vitrectomy with ILM peeling was carried out for refractory DME 6 months prior to their inclusion in this study.

### 3.2. Operative Complications

Intraoperative complications were identified in three eyes. An iatrogenic macular hole occurred in two eyes (7.1%) during subretinal BSS injection, but postoperatively, the hole was found to be closed with improvement of BCVA (Video 1). In another case, an iatrogenic break occurred in the nasal retina during injection of the dual stain. Endolaser was applied, and the patient was instructed to attain a prone position for two days.

### 3.3. Postoperative Findings

The cases had a mean follow-up period of 10.6 ± 1.1 months postoperatively. The mean preoperative CMT was 496.07 ± 88.7 *μ*m, while the postoperative mean CMT decreased to 335 ± 67 *μ*m, when measured 4 weeks postoperatively. The mean CMT further dropped with subsequent OCT measurements and reached a mean of 274.1 ± 31.6 *μ*m at the final follow-up visit for all included eyes (*p* = 0.029).

**Figure 1:**
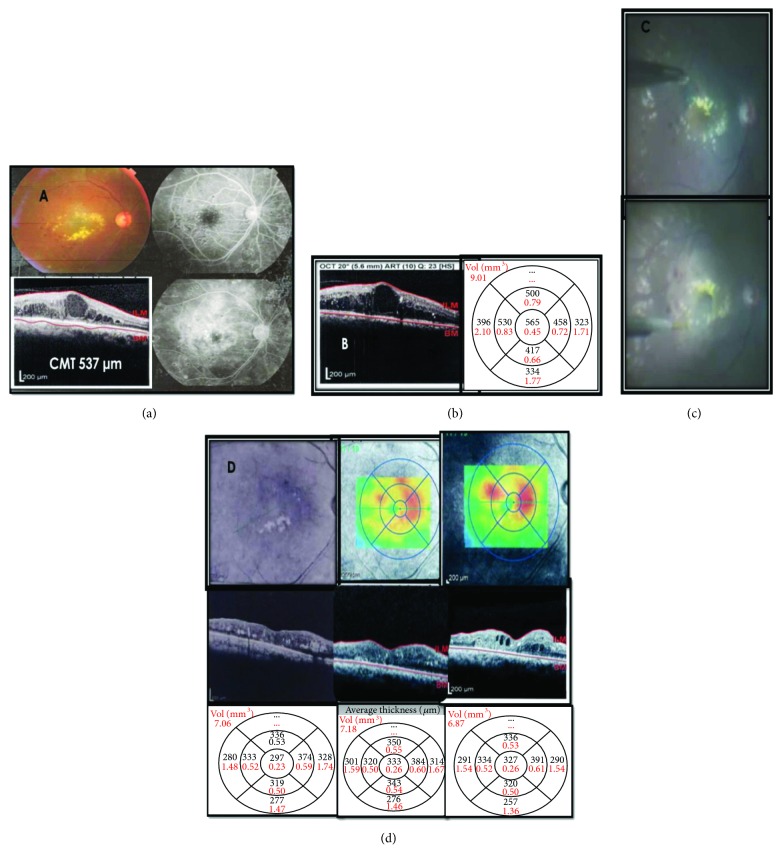
(a) Preoperative color fundus photo and FA showing diffuse DME with foveal hard exudate accumulation, CMT by OCT measuring 537 microns after 9 IVB injections over 1 year, BCVA measuring 0.1. (b) OCT after 3 IV triamcinolone (TA) injections 3 months apart with CMT measuring 565 microns and no improvement in BCVA. (c) Upper photo showing ILM peeling after dual stain application, while lower phot showing submacular BSS injection. (d) Red free showing significant decrease in amount of hard exudates 1 month postoperation, with drop of CMT to 297 microns and BCVA improvement to 0.3. Middle OCT with the thickness map showing recurrence of DME measuring 333 microns 6 months postoperation. The right-hand side OCT image and thickness map after 2 IVTA injections 2 months apart with slight CMT improvement of 327 microns while regaining a BCVA of 0.3 which was measured 10 months postoperation.

Six eyes (21.4%) suffered from recurrence of their edema defined as increase in CMT by more than 10% of the least thickness attained during the period of follow-up, with concomitant drop of at least one line of BCVA. Intravitreal triamcinolone (IVTA) (once in 2 eyes and twice in 4 eyes) was given to treat these recurrences. All these eyes showed improvement of CMT and BCVA after IVTA and regained the postintervention parameters (Figure 1).

In all operated 28 eyes, the preoperative mean ± SD BCVA in decimal form was 0.2 ± 0.11, while at the final follow-up visit, the mean ± SD BCVA improved to 0.45 ± 0.2 (*p* = 0.000019). No improvement occurred postoperatively in the ellipsoid zone integrity in all eyes even in those with complete resolution of edema. Despite this finding, BCVA did improve in eyes with edema resolution to different extents. As for the ELM, postoperative 16 eyes (57.1%) showed continuous ELM with resolution of edema.

### 3.4. Subgroup Analysis

**Figure 2:**
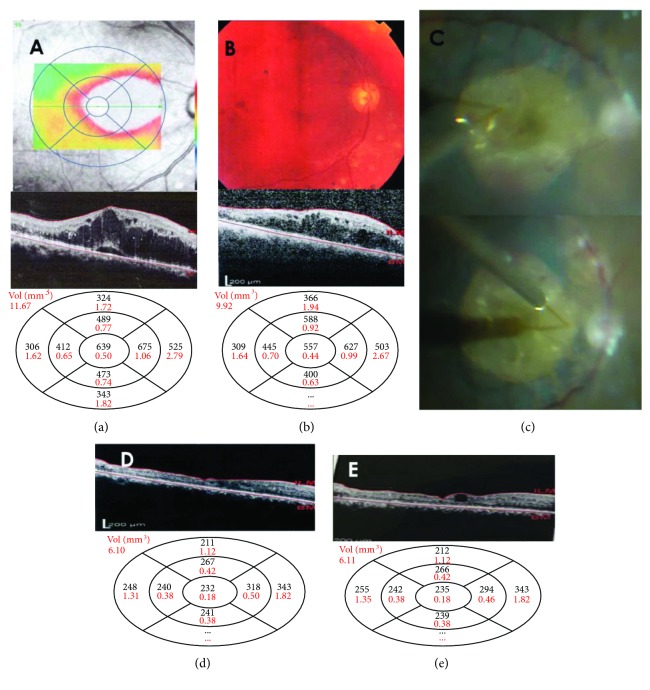
(a) Right eye: preoperative red free color-coded map showing marked DME with cystoid and neurosensory detachment shown in the OCT image, disruption of both ellipsoid zone, and ELM, with CMT measuring 639 microns after 8 IVR injections and 3 IVA injections over 1 year with BCVA equals 0.06. (b) Color fundus photo and OCT image of the same eye after two IVTA injections with CMT improving to 557 microns, but BCVA remained at 0.06. (c) Subretinal BSS injection after ILM peeling done at 2 different sites to cover the entire area of edema. (d) OCT image showing complete resolution of edema 4 weeks postoperatively with a CMT of 232 microns and BCVA of 0.16. The ellipsoid zone and ELM integrity were not regained. (e) CMT measured 10.2 months later equals 235 microns with stable BCVA.

**Figure 3:**
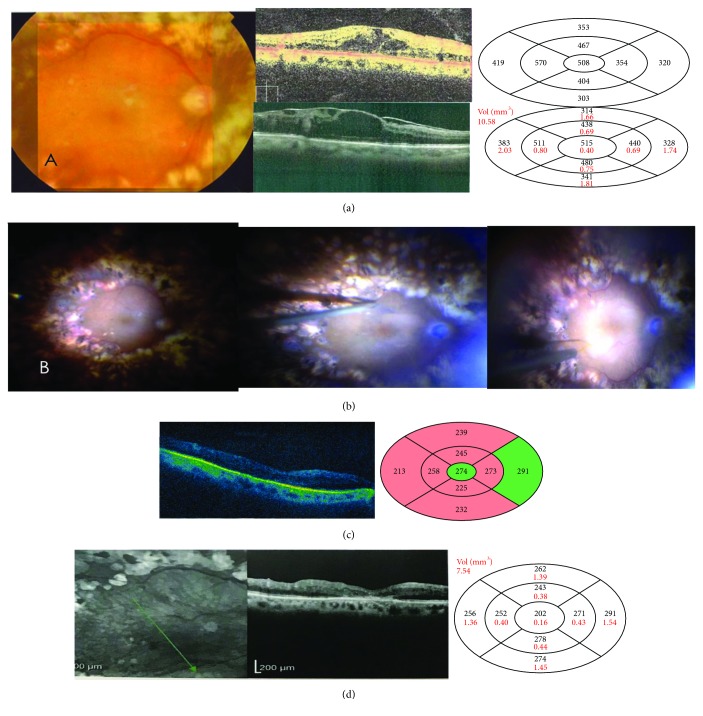
(a) Color fundus photo of a 59-year-old female who had vitrectomy done for refractory DME after failure of anti-VEGF (10 IVB and 3 IVA) to improve the edema. Upper OCT image and map showing CMT of 508 microns a year after the vitrectomy with BCVA of 0.05, totally disrupted ellipsoid zone and ELM. Lower OCT image and thickness map after 3 IVTA injections 3 months apart as a trial to improve the edema, CMT measuring 515 microns without VA gain and appearance of an ERM. (b) During surgery, ILM peeling was reattempted, and submacular BSS was injected to cover the whole area of the edema. (c) OCT of the macula 1 month postoperatively shows resolution of the edema with CMT 274 microns and BCVA of 0.1. (d) Red free photo 9.5 months postop. with thickness dropped further to 202 microns and BCVA still 0.1, probably due to the marked ELM and ellipsoid zone disruption.

Cases were divided into group 1 which included eyes where vitrectomy was not attempted as a solution for refractory DME (Figure 2) and group 2 which included eyes with a history of vitrectomy for more than 6 months (Figure 3).  Table 1 shows the pre- or postoperative characteristics of the two groups.

**Figure 4:**
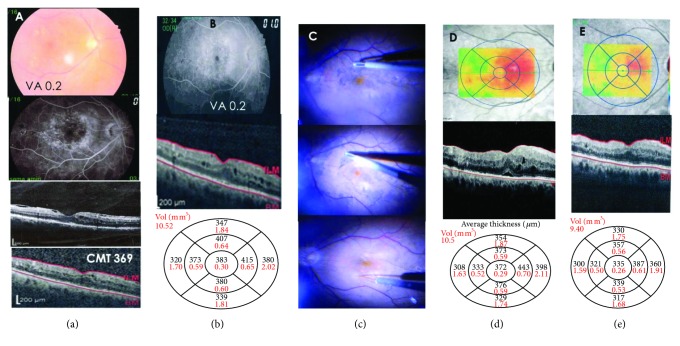
(a) Color fundus photo and late FA image of a male 53 years of age with type 2 DM, suffering from refractory DME with VMA with a CMT of 369 microns after 8 IVR injections over the past 9 months. BCVA recorded was 0.2. (b) Diffuse DME shown in a late FA image with CMT of 383 after shifting to IVA for three consecutive injections. (c) Upper snap shot during removal of the fine ERM stained with the dual stain, middle image showing ILM peeling, while the lower photo was taken during submacular BSS injection. (d) Red free with color-coded map showing slight CMT improvement 4 weeks postoperatively reaching 372 microns. (e) Eight months postoperation with edema reaching 335 without additional treatment and BCVA improved to 0.8.

Eyes included were also divided into group a (with normal vitreomacular interface) and group b (vitreoretinal abnormalities present in the form of ERM, Figures 3 and 4).  Table 2 shows a comparison between groups a and b.

## 4. Discussion

Despite all the pharmacological and surgical interventions currently utilized for refractory DME, the results for many cases are disappointing. This led to the introduction of the planned foveal separation with submacular BSS injection with favorable results [15]. In addition to its success in cases in which all other treatment protocols failed, a rapid edema resolution was noticed. The technique was associated with intact ELM and ellipsoid zone on OCT and better visual outcomes which was clearly depicted in previous studies tackling this point [13, 14, 18–20]. Yet, this technique had not been previously attempted in vitrectomized eyes and in those with ERM.

The refractory edema responded better with this technique than with conventional vitrectomy with or without ILM peeling. This was shown by Ulrich et al., who found that there was no significant change in CMT at 1 and 3 months after conventional vitrectomy, (*p* = 0.91, 0.29) or in visual acuity (*p* = 0.69, 0.21). However, it was not until 6 months postoperatively that the CMT had significantly decreased (*p* = 0.03) and the visual acuity showed improvement (*p* = 0.0) [19]. Similarly, the Diabetic Retinopathy Clinical Research Network reported that 3 months after vitrectomy, the decrement in CMT was only 160 *μ*m [7]. Likewise, Yamamoto et al. observed that although the CMT decreased by 140 *μ*m 1 week after surgery, it took 4 months for the CMT to drop below 300 *μ*m [9].

The current study demonstrated a more rapid and significant decrease in CMT: by 163.9 ± 32.6 *μ*m after 4 weeks and 227.01 ± 80.01 *μ*m at the final visit (10.6 ± 1.2 months) in group a and by 147.97 ± 16.2 *μ*m after 4 weeks and 203.17 ± 70.4 *μ*m at the final visit (10.5 ± 0.5 months) in group b, but this difference was not statistically significant (*p* = 0.645). Likewise, BCVA improved in group a from a mean of 0.2 ± 0.11 preoperatively to a mean of 0.44 ± 0.2 postoperatively and from a mean of 0.217 ± 0.11 preoperatively to 0.5 ± 0.22 postoperatively in group b. These values were again not statistically significant. These results indicate that the planned foveal detachment technique works like an adjunctive step to conventional vitrectomy to speed up the resolution of DME and improve BCVA, regardless of the vitreomacular interface state before the surgery.

The rapid resolution of macular edema by the planned foveal detachment technique was noticed to be more in surgically naïve DME patients (group1) measuring 239 *μ*m at the final follow-up visit than in group 2 eyes, subjected previously to both anti-VEGF and conventional vitrectomy, reaching 170.8 *μ*m at the final follow-up visit. This difference in outcome was statistically significant (*p* = 0.019).

As regards the visual acuity, the preoperative BCVA in group 1 (surgically naïve eyes) was 0.16 ± 0.07, which improved to a mean of 0.34 ± 0.09, while in group 2 (eyes with previous vitrectomy), the preoperative BCVA was 0.37 ± 0.14, which improved to a mean of 0.7 ± 0.16 postoperatively. This was not statistically significant (*p* = 0.185). So, although there was a significant difference in the mean CMT between the two groups (1 and 2), the mean BCVA postoperatively did not differ significantly. This might be explained by the fact that the chronicity of the edema in both groups was a limiting factor against marked BCVA improvement despite the greater improvement in CMT. This was obvious in group 1 where similar improvement in postoperative BCVA occurred despite a marked drop of CMT in relation to group 2.

The superiority of planned foveal detachment may be explained by multiple factors according to Morizane et al. These include facilitation of egress of edema fluid from the retina to the choroid by reducing both the oncotic pressure and viscosity of the subretinal fluid as well as the wash out of inflammatory cytokines and migratory cells above the RPE. Both mechanisms might be responsible for activation of the RPE to pump fluid from the retina to the choroid. Since these mechanisms could be effective within hours or days of surgery, they were consistent with their observations of rapid complete resolution of the macular edema after surgery [15].

In the present study, it is also notable that the resolution of DME continued for at least 10 months without additional treatment in most cases (22 eyes, 78.6%). This long-term effect may be explained by the fact that marked and rapid improvement in the retinal environment, due to drainage of the edema fluid, breaks the vicious cycle of ischemia-vascular hyperpermeability-chronic inflammation-ischemia seen in diabetic patients [15].

During the surgical procedure for the planned foveal detachment technique, special attention is needed to avoid an iatrogenic macular hole or injuries to Bruch's membrane during the subretinal injection of BSS. Therefore, Morizane et al. used a viscous fluid-control system (Alcon Laboratories, Fort Worth, TX, USA) with a low injection pressure to regulate the speed of subretinal injection [15].

In the present study, a similar maneuver was used for subretinal fluid injection. Still, 2 cases (7.1%) suffered iatrogenic holes during injection. However, the postoperative follow-up revealed closure of the macular holes with improvement of the final visual acuity in these cases. Even without submacular saline injection, the risk of macular hole induction exists, as Grigorian et al. reported an incidence of 2% with conventional vitrectomy for DME [21].

Most of the cases included in the current study have had DME for more than a year, with significant ellipsoid zone (EZ)—previously called the photoreceptor inner segment/outer segment (IS/OS) junction—disruption in 15 eyes (53.6%). In these cases, ellipsoid zone disruption neither improved nor worsened postoperatively. Despite this, the CMT, BCVA and, to a certain extent, the ELM continuity improved after resolution of edema postoperatively. A similar conclusion was drawn by Chhablani et al., where the strongest clue for vision improvement was preoperative damage to the ELM (*p* = 0.0277) compared to the IS/OS junction (*p* = 0.03) [22].

In conclusion, vitrectomy with planned foveal detachment technique appears to be a promising solution for DME cases that is resistant to all other forms of treatment (repeated anti-VEGF, Cst injections, and even conventional vitrectomy with ILM peeling) with rapid and efficient edema resolution in those resistant eyes. CMT was better in eyes where conventional vitrectomy was not attempted. Moreover, cases with VMA resistant to pharmacotherapy was shown to respond well to this technique.

The current study is limited by its uncontrolled design and small sample size. Further randomized controlled clinical studies involving a larger number of patients with longer duration of follow-up are needed to define the exact role of this procedure in the management of DME.

## Figures and Tables

**Table 1:** Pre- and postoperative characteristics of groups 1 and 2.

Variable studied	Group 1	Group 2	*p* value
Number of eyes in each group	21	7	
Age mean ± SD (years)	53.38 ± 8.2	52.7 ± 3.4	
OCT findings			
Presence of neurosensory detachment preoperatively	9 (42.9%)	4 (57.1%)	0.51
Presence of VMA preoperatively	3 (14.3%)	3 (42.9%)	0.11
Intact ellipsoid zone	10 (47.6%)	3 (42.9%)	0.827
Preop. continuous ELM	9 (42.9%)	3 (42.9%)	1.0
Postop. continuous ELM	13 (69.9%)	3 (42.9%)	0.3
Preoperative CMT mean ± SD (*μ*m)	521.3 ± 83.6	420.2 ± 56.3	
Postoperative CMT (final visit) mean ± SD (*μ*m)	282.3 ± 20.8	249.4 ± 45.7	0.019^∗^
Preoperative injection history			
Bevacizumab + aflibercept	10 (47.6%)	3 (42.9%)	0.11
Ranibizumab + aflibercept	12 (57.1%)	4 (57.1%)	1.0
CST after anti-VEGF failure	8 (38.1%)	4 (57.1%)	
BCVA (decimal form)			
Preoperative	0.16 ± 0.07	0.34 ± 0.09	
Postoperative (final visit)	0.37 ± 0.14	0.7 ± 0.16	0.185^∗^
Recurrence of edema within FU period	6 (28.6%)	0 (0.0%)	0.1
Follow-up period in months	10.57 ± 1.1	10.86 ± 1.2	

^∗^Mann–Whitney test.

**Table 2:** Characteristics of group a (normal vitreomacular interface) and group b (vitreoretinal abnormalities present).

Variable studied	Group a	Group b	Significance (2-tailed)
Number of eyes in each group	22	6	
Age	52.82 ± 7.6	54.5 ± 6.3	
Previous vitrectomy attempted	4 (18.2%)	3 (50.0%)	0.288
Follow-up in months	10.68 ± 1.2	10.5 ± 0.5	
OCT characteristics of the 2 groups			
Preoperative mean ± SD CMT (*μ*m)	497.6 ± 93.1	490.3 ± 77.3	
CMT at 4 weeks	333.7 ± 69.7	342.33 ± 61.1	
Final CMT	270.5 ± 33.9	287.1 ± 16.6	
CMT improvement	227.01 ± 80.01	203.17 ± 70.4	0.645^∗^
Intact ELM at final visit	14 (63.6%)	2 (33.3%)	0.354
BCVA in decimal form			
Preoperative	0.2 ± 0.11	0.217 ± 0.11	
Final	0.44 ± 0.2	0.5 ± 0.22	
Lines of improvement	3.82 ± 29	3.67 ± 1.21	0.883^∗^
Recurrence of macular edema	6 (27.3%)	0	0.289
Complications			
Macular hole	1 (4.5%)	1 (4.5%)	0.529
Iatrogenic break	1 (16.7%)	0 (0.0%)	0.435

^∗^Mann–Whitney.

## Supplementary Materials

Supplementary Material Video 1: edited video showing the ILM peeling in a surgically naïve case with a refractory DME in spite of 9 IVB injections during the course of 1 year and 3 IV triamcinolone (TA) injections 3 months apart, BCVA measuring 0.1. Secondary iatrogenic macular hole occurred during submacular BSS injection. Fluid-air exchange at the end of the surgery. Video 2: edited video showing a case with fine EMM and refractory DME in spite of repeated ranibizumab injections and 3 aflibercept injections along the course of 8 months, the EMM that was peeled using an end-gripping 23-gauge forceps after staining with dual stain, subretinal injection of BSS was attempted as mentioned above. Fluid air exchange was then done. Video 3: edited movie for an eye with persistent DME despite previous vitrectomy, staining was also done under air to ensure ILM removal above the entire area involved in the edema process and proper peripheral vitreous trimming before attempting subretinal BSS injection was done. Air was left as a tamponade at the conclusion of surgery.
